# Diabetes mellitus medication use and catastrophic healthcare expenditure among adults aged 50+ years in China and India: results from the WHO study on global AGEing and adult health (SAGE)

**DOI:** 10.1186/s12877-016-0408-x

**Published:** 2017-01-11

**Authors:** Shingai Douglas Gwatidzo, Jennifer Stewart Williams

**Affiliations:** 1 Umeå International School of Public Health, Unit of Epidemiology and Global Health, Department of Public Health and Clinical Medicine, Faculty of Medicine, Umeå University, SE-90185 Umeå, Sweden; 2 Unit of Epidemiology and Global Health, Department of Public Health and Clinical Medicine, Faculty of Medicine, Umeå University, SE-90185 Umeå, Sweden; 3 Research Centre for Gender, Health and Ageing, Faculty of Health, University of Newcastle, New Lambton Heights, Newcastle, NSW 2305 New South Wales Australia

**Keywords:** Non communicable diseases, NCDs, Out-of-pocket, OOP, Ageing, Aging, Developing countries, Low- and middle-income countries, Universal healthcare coverage, UCC, Financing, Impoverishment, Medicines

## Abstract

**Background:**

Expenditure on medications for highly prevalent chronic conditions such as diabetes mellitus (DM) can result in financial impoverishment. People in developing countries and in low socioeconomic status groups are particularly vulnerable. China and India currently hold the world’s two largest DM populations. Both countries are ageing and undergoing rapid economic development, urbanisation and social change. This paper assesses the determinants of DM medication use and catastrophic expenditure on medications in older adults with DM in China and India.

**Methods:**

Using national standardised data collected from adults aged 50 years and above with DM (self-reported) in China (*N* = 773) and India (*N* = 463), multivariable logistic regression describes: 1) association between respondents’ socio-demographic and health behavioural characteristics and the dependent variable, DM medication use, and 2) association between DM medication use (independent variable) and household catastrophic expenditure on medications (dependent variable) (China: *N* = 630; India: *N* = 439). The data source is the World Health Organization (WHO) Study on global AGEing and adult health (SAGE) Wave 1 (2007–2010).

**Results:**

Prevalence of DM medication use was 87% in China and 71% in India. Multivariable analysis indicates that people reporting lifestyle modification were more likely to use DM medications in China (OR = 6.22) and India (OR = 8.45). Women were more likely to use DM medications in China (OR = 1.56). Respondents in poorer wealth quintiles in China were more likely to use DM medications whereas the reverse was true in India. Almost 17% of people with DM in China experienced catastrophic healthcare expenditure on medications compared with 7% in India. Diabetes medication use was not a statistically significant predictor of catastrophic healthcare expenditure on medications in either country, although the odds were 33% higher among DM medications users in China (OR = 1.33).

**Conclusions:**

The country comparison reflects major public policy differences underpinned by divergent political and ideological frameworks. The DM epidemic poses huge public health challenges for China and India. Ensuring equitable and affordable access to medications for DM is fundamental for healthy ageing cohorts, and is consistent with the global agenda for universal healthcare coverage.

## Background

In all parts of the world people are living longer, overall health has improved, and average income levels are rising. Yet despite major social and economic advancements, additional years of life are not always lived in good health. Economic development is associated with a number of social and demographic changes, covering urbanisation and the adoption of unhealthy lifestyle behaviours, including lack of physical exercise, tobacco use and excess alcohol consumption. These activities can lead to metabolic and physiological changes, such as high blood pressure, obesity, raised blood glucose and elevated cholesterol - all of which are risk factors for non-communicable diseases (NCDs). In recent decades the main causes of death and disability have shifted away from infectious diseases with NCDs now responsible for about 70% of premature mortality [1, 2]. At the Political Declaration on the Prevention and Control of NCDs in 2011, world leaders targeted four major NCD global priorities - cardiovascular disease, cancer, chronic respiratory diseases and diabetes mellitus (DM) [3].

China and India are the world’s two most populous countries with populations of 1.4 and 1.3 billion respectively [4]. More than 30% of adults aged over 50 now live in either country with this proportion expected to approach 40% by 2050. China and India hold prominent positions at global and regional levels and both countries are experiencing economic growth, demographic ageing, urbanisation and changes in population health. These policy elements alone warrant comparisons to better understand and project global disease burdens. Yet of particular interest are the differing trajectories of economic growth and epidemiological transition occurring in the two population superpowers [5–11].

China’s rapid economic growth has led to extraordinary increases in real living standards, improved access to government-funded healthcare and a decline in poverty [12]. India’s economic growth has been much slower, poverty rates remain high and the majority of the population do not have access to affordable healthcare [13]. Both countries are experiencing altered morbidity burdens due to increased life expectancies. China has made good progress with regard to successful infectious disease control, but now faces a rising epidemic of NCDs. India is experiencing a double burden of both communicable and NCDs [5, 6, 9]. This study compares factors associated with DM, medication use and catastrophic healthcare expenditure in China and India.

Diabetes mellitus (diabetes) increases the risk of serious morbidity and premature death from cardiovascular complications [14]. The condition occurs either when the pancreas does not produce enough insulin (type 1) or when the body cannot use the insulin it produces effectively, resulting in elevated plasma glucose levels (type 2) [15]. Over 90% of people with DM have type 2. This can be delayed or prevented by behaviour change, such as ceasing tobacco smoking, adhering to a healthy diet, and engaging in physical activity. Medications for DM are on the World Health Organization (WHO) list of essential medicines. People with type 1 DM cannot survive without injections of insulin. A number of medications are available for people with type 2 DM. People with all types of DM can also require medications for blood pressure and cholesterol control [16].

Diabetes has been described as one of the medical emergencies of the 21st century [16]. In 2015, the International Diabetes Federation (IDF) estimated that worldwide about 415 million adults aged 20–79 years (about 8.8%) had DM and that the condition accounted for 12% of global healthcare expenditure. The IDF predicts that, if current trends continue, by 2040 one in ten adults will have DM [16].

Current estimates are that about 75% of people with DM live in low- and middle-income countries (LMICs) [16]. In 2015 China had the world’s largest population of adults (aged 20–79 years) with DM at 109.6 million. India ranked second with 69.2 million adult cases [16]. The age-adjusted DM prevalence in 20–79 year olds at 9.8% in China and 9.3% in India. Diabetes poses enormous public health challenges for both countries [16]. Between 2015 and 2040, China is expected to experience a 37.5% increase in the numbers of adults (20–79 years) with DM (to 150.7 million). On current projections, the absolute numbers of adults with DM in 2040 will be larger in China than in India (150.7 million compared with 123.5 million). However India is expected to experience a 117.8% increase in the numbers of adults with DM between 2015 and 2040 (from 69.2 million to 123.5 million) [16].

Diabetes impacts disproportionately on those who are older and socially and economically vulnerable [16–19]. In 2015, the United Nations General Assembly adopted the 2030 Agenda for Sustainable Development [20, 21]. Countries agreed to: take action to reduce premature mortality from DM and other NCDs by one-third; achieve universal healthcare coverage (UHC), and provide access to affordable essential medicines. See http://www.idf.org/action-on-diabetes/sdgs.

One of the impediments to achieving UHC is reliance on out-of-pocket (OOP) payments. These are fees paid by the patient to the provider at the time of the service [22] and they can include payments made for consultations, procedures and medicines [23, 24]. It is estimated OOP payments comprise an overwhelming majority of household medical expenditure in developing countries and that about one third of people in developing countries are unable to afford essential medicines on a regular basis [25, 26].

According to the World Bank, between 2011 and 2015, OOP expenditure as a percentage of private expenditure on health, was 72.3% in China and 89.2% in India [27]. Catastrophic healthcare expenditure refers to situations where households make OOP payments for healthcare above a reasonable proportion of their income [28] one of the consequences of which is decreased spending on food and other essentials [29]. In a study which described the magnitude and distribution of OOP payments and catastrophic expenditures in Asia, China and India were identified as relying heavily on OOP payments and having a high incidence of catastrophic payments for healthcare [26]. Catastrophic healthcare expenditure was estimated at 13.0% in China in 2008 [30]. Research in India suggests that in addition to having DM, people living in rural areas [31] and having lower incomes, incur higher OOP payments [32].

The authors of a literature review of NCD costs in LMICs concluded that NCDs impose a disproportionate financial burden on poorer households. Expenditure on medications and treatments for DM comprise a major source of household expenditure on healthcare [33]. Another review on this topic showed that in LMICs, 6–11% of the total population would be impoverished if they had to purchase even low-priced generic medications for DM [34]. A study which analysed the findings of a national survey conducted in China in 2008 found that healthcare costs were higher for people with DM compared with people with normal glucose tolerance [35].

Given the global debates about UHC and healthcare financing, and the increasing prevalence of NCDs alongside ageing populations, there is now, more than ever, a need to develop and implement social protection policies as a way of improving financial risk protection for healthcare. This is particularly important for LMICs, where the financial costs are largely borne by individuals and households [26, 33, 36]. Some research has looked into the financial impact of NCDs in high-income countries and the evidence base for LMICs is slowly amassing [33, 37]. An analysis of data from in 35 LMICs in the World Health Surveys (2002–2003) showed that, regardless of insurance coverage, diabetic individuals (aged > =18 years) were more likely to experience catastrophic medical spending [29].

Globally there is major public health concern about the health and economic consequences of DM with attention directed to two countries in the Asia-Pacific region which are home to more than 30% of the world’s population [4, 16, 21, 38]. This study unpacks factors associated with medication use and catastrophic expenditure on medications among adults with self-reported DM in China and India. The China India comparison will provide insights into how health system characteristics might differently impact on catastrophic healthcare expenditure in households in which there are people with DM.

The aims are to assess the determinants of medication use and catastrophic expenditure on medications in adults aged 50 years and above who self-reported DM in national surveys conducted in China and in India. The research questions are as follows. Among older adults in China and India who report having DM, what factors are associated with medication use? Are households in which there are people with DM more likely to incur catastrophic expenditure on medicines? To our knowledge, this is the first study of its type. In addition to informing global policies and interventions for people with DM in China and India, the findings draw attention to the need to address healthcare financing of essential medicines.

## Methods

### Data collection

The data source for this study is the WHO Study on global AGEing and adult health (SAGE) Wave 1 (2007–2010). WHO-SAGE is a longitudinal study of health and ageing in six LMICs - China, Ghana, India, Mexico, Russia and South Africa. This study covers China and India only. The cohorts comprise nationally representative samples of “older adults” aged 50 years and above. This age cut-point is consistent with the WHO definition of younger and older adults in LMICs [39]. Data were collected using structured household and individual questionnaires administered in face-to-face interviews conducted in local languages. The individual questionnaire includes information on sociodemographic factors, health states and behaviours and medication use. The household questionnaire includes information on dwelling characteristics, asset ownership, income and expenditure.

WHO-SAGE employed a stratified random sampling strategy in all countries with households as the final sampling units. The strata ensure representation of a range of living conditions and urban and rural localities in each country. The probability proportional to size sampling method was used to select primary sampling units (PSUs) within the strata - towns in China and villages in India - and households were selected randomly within PSUs Household-level analysis weights and person-level analysis weights were calculated for each country and post-stratification weights are used to adjust for age and sex distributions and non-response [40]. Data from WHO-SAGE are in the public domain and details are reported elsewhere [39].

### Study variables derived from the individual questionnaire

The study population was conditioned on confirmatory responses to the question: Have you ever been diagnosed with diabetes (high blood sugar)? *(Not including diabetes associated with a pregnancy)* in the individual questionnaire. Those who answered “yes” were categorised according to whether they had been taking insulin or other blood sugar lowering medications either 1) in the last two weeks or 2) in the last twelve months. The binary medication variable enables a two-group comparison between past-year DM medications users and others who reported non-use of DM medications.

Socio-demographic variables are sex, age, residence, marital status, educational status and wealth status. Sex is male or female. The age categories are 50–59 years versus 60–69 years versus 70–79 years versus 80+ years. Residence is urban or rural. Marital status is never married versus married/cohabiting, versus divorced or widowed. Educational status is primary school or less, versus secondary or high school, versus university or higher.

Health behaviour variables are body mass index (BMI), nutrition and physical activity. The BMI variable is derived from physical measurements of weight in kilograms (kg_s_) and height in metres (m_s_). The WHO guidelines on appropriate BMI for Asian populations [41] are used to derive the categories high BMI (> = 30 kg/m^2^) versus low BMI (<30 kg/m^2^). The nutrition variable is derived from reported daily intake of fruit and vegetables (> = 5 servings daily) versus insufficient intake of fruit and vegetables [42, 43]. Physical activity is measured using the Global Physical Activity Questionnaire [44, 45]. The classification is low versus moderate versus high. The lifestyle modification variable classifies “yes” or “no” responses to the question: have you been following a special diet, exercise regime or weight control program for diabetes during the last 2 weeks? *(As recommended by health professional).*

### Study variables derived from the household questionnaire

Wealth status is derived from information on dwelling characteristics (e.g., cooking oil, floor and roof types), ownership of durable goods (e.g., radio, car) and access to basic services (e.g., electricity, clean water and sanitation). Principal Components Analysis was used to generate weights from which raw continuous scores were derived. These scores were transformed into wealth quintiles which are included in the individual questionnaire dataset. Here quintile 1 includes individuals in the wealthiest or richest households and quintile 5 includes individuals in the poorest or least wealthiest households [46, 47]. The quintiles were set in the original data and therefore the use of survey sampling weights modify this distribution. Owing to small cell sizes the poorest two wealth quintiles were merged for the analysis of catastrophic expenditure.

Catastrophic healthcare expenditure on medications is a binary variable estimated by summing mandatory and voluntary expenditures reported for medications. There is discussion in the literature about appropriate cut points for catastrophic healthcare expenditure [28]. Using evidence from other studies of healthcare expenditure on medications in LMICs, catastrophic expenditure is defined as > 40% of reported household income spent on medications [33].

The household financial status variable was derived from responses to the question: Would you say your household's financial situation is (either): very good; good; moderate; bad, or very bad? Due to small numbers in the cells the “very good” and “good” categories were combined as were the “very bad” and “bad” categories.

Information in the household questionnaire is also used to derive a variable that describes the educational status of the household head as: no schooling versus primary school or less versus secondary/high school versus university or higher.

### Data preparation and study sample

**Fig. 1:**
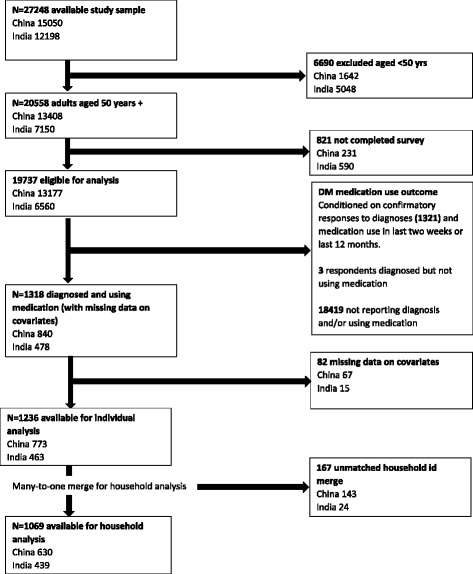
Derivation of Study Sample

Figure 1 shows the derivation of the study samples in China and India. The available study populations of SAGE Wave 1 respondents was 27,248 of whom 15,050 were in China and 12,198 in India. Only respondents aged 50 years and above who completed the SAGE surveys were included. Eligibility for the study sample also required that respondents: 1) reported having been diagnosed with DM or high blood sugar, not including DM associated with a pregnancy, and 2) had non-missing responses to the questions asked about DM medications use, either in the past year or past two weeks, and on any other study variables in the individual questionnaire.

After satisfying the above criteria, two study samples from China (*N* = 773) and India (*N* = 463), were used for the analysis of DM medication use. A many-to-one merge was performed between the individual and household questionnaire datasets. Records with missing data on household study variables were excluded, giving study samples of 630 in China and 439 in India for the analysis of household catastrophic healthcare expenditure on medications.

### Ethics statement

The SAGE study was approved by the Ethics Review Committee, World Health Organization, Geneva, Switzerland; the Ethics Committee, Shanghai Municipal Centre for Disease Control and Prevention, Shanghai, China, and the Institutional Review Board, International Institute of Population Sciences, Mumbai, India. Approval covered all procedures undertaken as part of the study. All participants gave written informed consent.

### Statistical analysis

Chi-squared tests of statistical significance compare socio-demographic and health behavioural characteristics by DM medication use in China and India. Multivariable logistic regression describes association between individual socio-demographic and health behavioural characteristics and the dependent variable, DM medication use. The selection of independent variables was informed by the literature on health seeking behaviour and health service utilization in developing countries [48, 49].

Chi-squared tests of significance compare household characteristics by catastrophic household healthcare expenditure on medications. Multivariable logistic regression describes association between DM medication use (independent variable) and catastrophic household healthcare expenditure on medications (dependent variable) while adjusting for possible confounding by household socioeconomic characteristics (residence, wealth quintile, financial status, and the educational status of the household head).

The regression models were tested for multicollinearity using the variance inflation factor (VIF) statistic. All analyses included survey sampling weights. The statistical software used here was STATA version 13 (Stata Corp, Lakeway Dr, College Station, TX 77845, USA).

## Results

Table 1 compares socio-demographic and health behavioural characteristics by DM medication use in China and India. Almost 90% of people with DM in China were using DM medications compared with 71% in India. Significantly higher proportions of medication users reported lifestyle modification in China (81.2%. versus 40.6%¸ *p* < 0.01) and India (69.9% versus 33.1%, *p* < 0.05). In China a higher proportion of medication users were females (59.9%) than non-users (50.6%). In India these proportions of users versus non-users were 39.1 and 32.6% respectively. There were statistically significant differences in household wealth by DM medication use only in India. In India, over 35% of non-DM medication users were in the poorest two household wealth quintiles compared with about 11% of non-users (*p* < 0.05). In China, there were significant differences by BMI in DM medications use (*p* < 0.05). Over 52% of users were in the high BMI group compared with 40.7% of non-users. A significantly (*p* < 0.05) higher proportion of DM medication users compared with non-users reported high physical activity in China (32.6% versus 28.0%).

**Table 1:** Socio-demographic and health behavioural characteristics by diabetes medication use, adults aged 50+ years, China and India, SAGE Wave 1, 2007–2010

	China			India		
	No meds	Meds	Total	No meds (*N* = 134)	Meds (*N* = 329)	Total
	N (^a^ %)	N (^a^ %)	N (^a^ %)	N (^a^ %)	N (^a^ %)	N (^a^ %)
Overall	101 (13.1)	672 (86.9)	773 (100)	134 (28.9)	329 (71.1)	463 (100)
Lifestyle modification						
No	54 (59.4)***	138 (18.8)	192 (24.7)	104 (66.9)**	97 (30.1)	201 (39.9)
Yes	47 (40.6)	534 (81.2)	581 (75.3)	30 (33.1)	232 (69.9)	262 (60.1)
Sex						
Male	50 (49.4)*	275 (40.1)	325 (41.4)	73 (67.4)	180 (60.9)	253 (62.7)
Female	51 (50.6)	397 (59.9)	448 (58.6)	61 (32.6)	149 (39.1)	210 (37.3)
Age groups (years)						
50–59	33 (36.1)	185 (28.2)	218 (29.4)	53 (57.3)	135 (50.8)	188 (52.5)
60–69	33 (34.7)	235 (38.7)	268 (38.1)	48 (26.7)	117 (28.6)	165 (28.1)
70–79	30 (26.2)	204 (27.4)	234 (27.2)	24 (11.4)	65 (18.1)	89 (16.3)
80+	5 (3.1)	48 (5.8)	53 (5.4)	9 (4.6)	12 (2.5)	21 (3.1)
Residence						
Urban	71 (67.0)	522 (75.0)	593 (73.8)	64 (44.7)	172 (48.9)	236 (47.8)
Rural	30 (33.0)	150 (25.0)	180 (26.2)	70 (55.3)	157 (51.1)	227 (52.2)
Marital status						
Never married	1 (2.0)	2 (0.3)	3 (0.6)	1 (0.1)	2 (0.03)	3 (0.06)
Married/cohabitating	87 (87.5)	549 (83.5)	636 (84.1)	91 (82.8)	257 (84.3)	348 (83.9)
Divorced/widowed	13 (10.5)	121 (16.2)	134 (15.3)	42 (17.0)	70 (15.7)	112 (16.1)
Educational attainment						
University or higher	9 (5.9)	46 (6.7)	55 (6.6)	12 (9.6)	48 (15.6)	60 (14.0)
Secondary/High School	39 (38.1)	273 (40.2)	312 (39.9)	33 (38.8)	102 (33.3)	135 (34.8)
Primary school or less	53 (56.0)	353 (53.1)	406 (53.5)	89 (51.7)	179 (51.1)	268 (51.3)
Household wealth						
1 (Richest)	30 (28.8)	162 (28.1)	192 (28.2)	53 (37.1)**	147 (48.3)	200 (45.3)
2	27 (28.5)	181 (27.0)	208 (27.2)	31 (17.3)	99 (28.6)	130 (25.6)
3	21 (24.0)	152 (22.0)	173 (22.3)	21 (10.1)	47 (12.0)	68 (11.5)
4	15 (11.6)	108 (15.6)	123(15.0)	18 (29.2)	22 (7.2)	40 (13.1)
5 (Poorest)	8 (7.0)	69 (7.4)	77 (7.3)	11 (6.4)	14 (3.9)	25 (4.5)
BMI						
Low	62 (59.3)**	338 (47.6)	400 (49.3)	105 (67.9)	222 (68.5)	327 (68.3)
High	39 (40.7)	334 (52.4)	373 (50.7)	29 (32.1)	107 (31.5)	136 (31.7)
Nutrition						
Adequate	75 (82.2)	510 (79.2)	585 (79.7)	9 (4.4)	31 (9.3)	40 (8.0)
Inadequate	26 (17.8)	162 (20.8)	188 (20.4)	125 (95.6)	298 (90.7)	423 (92.0)
Physical activity						
High	26 (28.0)**	191 (32.6)	217 (31.9)	62 (63.1)	127 (44.9)	189 (49.8)
Moderate	28 (23.1)	232 (33.6)	260 (32.1)	31 (17.0)	99 (29.4)	130 (26.1)
Low	47 (48.9)	249 (33.8)	296 (36.0)	41 (19.9)	103 (25.7)	144 (24.1)

Pearson *χ* ^2^ tests undertaken for country comparisons. *p-value < 0.10; **p-value < 0.05; ***p-value < 0.01

^a^survey sampling weights used to give percentage estimates. Percentages may not sum due to rounding

Table 2 presents the multivariable logistic regression of socio-demographic and health behavioural characteristics by DM medication use in China and India. People who reported lifestyle modification were six times more likely to use DM medications in China (OR 6.22; 95% CI:3.80–10.20) and eight times more likely to use DM medications in India (OR 8.45; 95% CI:3.97–18.0). The odds of females using DM medications in China were almost 60% higher than for males (OR 1.56; 95% CI:0.99–2.47) although the result was significant only at *p* < 0.10. People in the poorer wealth quintiles in China were more likely to use DM medications whereas the reverse was true in India. For example, people in the second poorest wealth quintile in China were twice as likely to use DM medications (OR 2.13; 95% CI:0.86–5.26) although the result was not statistically significant. However in India the odds of people in the second poorest wealth quintile using DM medications were statically significant (*p* < 0.05) and 86% lower than the odds in the richest quintile (OR 0.14; 95% CI:0.03–0.69).

**Table 2:** Multivariable logistic regression of association between socio-demographic and health behavioural characteristics and diabetes medication use, adults 50+ years, China and India, SAGE Wave 1, 2007–2010

	China (*n* = 773)		India (*n* = 463)	
	Odds Ratio	95% CI	Odds Ratio	95% CI
Lifestyle modification				
No	1	Reference	1	Reference
Yes	6.22***	(3.80–10.2)	8.45***	(3.97–18.0)
Sex				
Male	1	Reference	1	Reference
Female	1.56*	(0.99–2.47)	1.06	(0.45–2.48)
Age groups (years)				
50–59	1	Reference	1	Reference
60–69	1.52	(0.84–2.72)	1.08	(0.50–2.30)
70–79	1.53	(0.69–3.36)	1.60	(0.62–4.17)
80+	3.44**	(1.02–11.5)	1.01	(0.45–7.06)
Residence				
Urban	1	Reference	1	Reference
Rural	0.85	(0.36–1.98)	1.29	(0.60–2.76)
Marital status				
Never married	1	Reference	1	Reference
Married/cohabitating	4.53	(0.38–54.2)	3.49	(0.32–37.7)
Divorced/widowed	6.16	(0.32–119.0)	1.97	(0.16–23.8)
Educational attainment				
University or higher	1	Reference	1	Reference
Secondary/High School	0.74	(0.15–3.74)	0.89	(0.26–3.00)
Primary school or less	0.45	(0.10–2.08)	1.60	(0.35–7.43)
Household wealth				
1 (Richest)	1	Reference	1	Reference
2	1.37	(0.69–2.80)	0.99	(0.45–2.19)
3	1.55	(0.72–3.36)	0.77	(0.28–2.13)
4	2.13	(0.86–5.26)	0.14**	(0.03–0.69)
5 (Poorest)	1.83	(0.62–5.40)	0.47	(0.09–2.30)
BMI				
High	1	Reference	1	Reference
Low	0.50***	(0.30–0.83)	1.26	(0.50–3.20)
Nutrition				
Adequate	1	Reference	1	Reference
Inadequate	1.18	(0.59–2.35)	0.88	(0.38–2.07)
Physical activity				
High	1	Reference	1	Reference
Moderate	1.02	(0.53–1.99)	3.09*	(0.94–10.2)
Low	0.44***	(0.25–0.76)	1.88	(0.86–4.11)

Mean Variance Inflation Factor (VIF) China–5.77

Mean Variance Inflation Factor (VIF) India = 4.65

Note: Survey sampling weights applied

95% CI = 95% Confidence Interval

*p-value < 0.10; **p-value < 0.05; ***p-value < 0.01

Table 3 compares household characteristics by catastrophic healthcare expenditure and shows that 16.8% of people with DM in China experienced catastrophic healthcare expenditure compared with 6.6% in India. In China significantly (*p* < 0.01) higher proportions households with catastrophic healthcare expenditure were in rural areas (49.3%) compared with people in households without catastrophic healthcare expenditure (29.1%). The data show an education gradient for catastrophic healthcare expenditure in China; over 67% of households that experienced catastrophic healthcare expenditure had household heads with primary school or less education compared with about 47% in households without catastrophic healthcare expenditure.

**Table 3:** Household characteristics by catastrophic health expenditure, adults 50+ years with diabetes, China and India, SAGE Wave 1, 2007–2010

	China (*n* = 630)		India (*n* = 439)	
	Non-catastrophic	Catastrophic	Non-catastrophic	Catastrophic
	N (^a^ %)	N (^a^ %)	N (^a^ %)	N (^a^ %)
Overall	524 (83.2)	106 (16.8)	410 (93.4)	29 (6.6)
Diabetes medication				
No	68 (14.1)	11 (12.8)	120 (26.8)	9 (24.8)
Yes	456 (85.9)	95 (87.2)	290 (73.2)	20 (75.2)
Lifestyle modification				
No	133 (24.5)	26 (29.7)	179 (39.4)	14 (41.3)
Yes	391 (75.5)	80 (70.3)	231 (60.6)	15 (58.7)
Residence				
Urban	398 (70.9)***	63 (50.7)	199 (47.6)	16 (32.6)
Rural	126 (29.1)	43 (49.3)	211 (52.4)	13 (67.4)
Household wealth				
1 (Richest)	131 (26.8)***	12 (8.8)	179 (46.1)	12 (29.5)
2	147 (27.9)	20 (25.4)	114 (24.7)	6 (31.3)
3	118 (22.9)	27 (27.9)	58 (11.5)	5 (11.5)
4 (Poorest 2 quintiles)	128 (22.4)	47 (37.9)	59 (17.8)	6 (27.7)
Household financial status				
Very good/Good	102 (18.9)	13 (12.9)	128 (33.0)	15 (52.4)
Moderate	331 (63.0)	66 (63.1)	202 (44.9)	8 (27.8)
Very bad/Bad	91 (18.1)	27 (24.1)	80 (22.2)	6 (19.9)
Educational attainment (household head)				
University or higher	45 (8.7)***	5 (3.3)	81 (21.5)	7 (10.6)
Secondary/High school	246 (44.1)	37 (29.6)	155 (41.4)	7 (35.5)
Primary school or less	233 (47.2)	64 (67.2)	174 (37.1)	15 (53.9)

Pearson *χ* ^2^ tests undertaken for country comparisons. *p-value < 0.10; **p-value < 0.05; ***p-value < 0.01

^a^survey sampling weights used to give percentage estimates. Percentages may not sum due to rounding

In the multivariable logistic (Table 4) DM medication use was not a statistically significant predictor of catastrophic healthcare expenditure in either country, although in China higher (*n* = 630) the estimated odds of catastrophic healthcare expenditure were 30% higher for DM medication users (OR 1.32; 95% CI:0.50–3.51). Compared with those in the highest (richest) wealth quintile in China, people in the two poorest quintiles were three and a half times more likely to be in households with catastrophic healthcare expenditure (OR 3.49; 95% CI:1.37–8.87). In India (*n* = 439) household financial status was significantly associated with catastrophic healthcare expenditure. People who reported very bad or bad compared with very good or good financial status, were less likely to be in households incurring catastrophic healthcare expenditure (OR 0.14; 95% CI:0.03–0.82).

**Table 4:** Multivariable logistic regression of association between DM medication use and household catastrophic health expenditure, adults aged 50+ with diabetes, China and India, SAGE Wave 1, 2007–2010

	China (*n* = 630)		India (*n* = 439)	
	Odds Ratio	95% CI	Odds Ratio	95% CI
Diabetes medication				
No	1	Reference	1	Reference
Yes	1.32	(0.50–3.51)	1.16	(0.29–4.62)
Lifestyle modification				
No	1	Reference	1	Reference
Yes	0.83	(0.48–1.45)	1.01	(0.32–3.14)
Residence				
Urban	1	Reference	1	Reference
Rural	1.62**	(1.01–2.61)	1.60	(0.28–9.24)
Wealth quintile				
1 (Richest)	1	Reference	1	Reference
2	2.38**	(1.21–4.66)	3.20	(0.42–24.19)
3	2.88***	(1.37–6.07)	3.01	(0.62–14.58)
4 (Poorest 2 quintiles)	3.49**	(1.37–8.87)	5.95*	(0.79–44.89)
Household financial status				
Very good/Good	1	Reference	1	Reference
Moderate	0.98	(0.45–2.14)	0.15***	(0.04–0.55)
Very bad/Bad	0.95	(0.40–2.26)	0.14**	(0.03–0.82)
Educational attainment (household head)				
University or higher	1	Reference	1	Reference
Secondary/High school	1.08	(0.36–3.26)	1.45	(0.18–11.45)
Primary school or less	1.68	(0.60–4.70)	2.64	(0.41–16.98)

Mean Variance Inflation Factor (VIF) China = 2.09

Mean Variance Inflation Factor (VIF) India = 1.56

Note: Survey sampling weights applied

95% CI = 95% Confidence Interval

*p-value < 0.10; **p-value < 0.05; ***p-value < 0.01

## Discussion

This study of adults aged 50 years and above in China and India with self-reported DM improves understanding of factors associated with DM medication use and catastrophic expenditure on medications and highlights divergent public health policies in these two rapidly developing populous countries. In China, healthcare costs are borne by the large government controlled public sector which is now responsible for the roll-out of UHC [50]. In India healthcare is financed by out-of-pocket (OOP) payments and private healthcare insurance with access to healthcare favouring higher socioeconomic groups [10]. Questions such as:*“*are there health system characteristics that make people more or less vulnerable to experiencing catastrophic expenditure?” need to become part of national policy debates. Only then can governments and policy-makers begin to explore ways of modifying and adjusting health system performance in order to protect households from catastrophic expenditure and impoverishment [18, 28].

Of those who self-reported DM, 87% in China and 71% in India, used medications for DM. A systematic review and meta-analysis of fifty-six studies (1979–2012) showed DM treatment rates of 93% in China [51]. In China’s National Diabetes and Metabolic Disorders Study, 81% of those who self-reported DM also reported using insulin or oral hypoglycaemic medicines [52]. A recently published study by the 10/66 Dementia Research Group found that 93% of people in urban China self-reported use of pharmacological therapies for DM [53]. In India the heterogeneity across and within states makes it difficult to generalise the data more broadly [54, 55]. Earlier research in India on DM showed that that 54% of people with DM were on oral hypoglycaemic agents, 22% on insulin and 20% on combination therapies [56]. More recently the Indian Council of Medical Research–India Diabetes (ICMR–INDIAB) Study found that the use of orally administered hypoglycaemic agents was 76% among respondents who self-reported DM [57].

Major healthcare reforms introduced by the Chinese government after 2002 included support and subsidisation of essential medicines for DM. Between 2001 and 2008 the percentage of OOP payments fell from 60 to 40% [52, 58, 59]. Between 50 and 80% of the cost of care for DM is met by the Chinese government [50, 60, 61].

Healthcare expenditure as a percentage of Gross Domestic Product in India is very low (4% in 2008) [13]. The private sector plays a major role in healthcare and only about 10% of medicines are subsidised by the public sector [62–64]. India has a rapidly expanding pharmaceuticals biotechnology market. In terms of the volume of global pharmaceutical production, India ranks fourth, yet 50 to 65% of the Indian population does not have access to essential medicines, compared with about 15% of the population in China [62].

Healthcare expenditure on medications was catastrophic for 17% in the China sample and 7% in the India sample. Although this indicates that 93% of people in the Indian sample did not experience catastrophic health expenditure on medications, it must be acknowledged that the majority of India’s population does not have access to quality affordable healthcare [13]. People in very poor households may have therefore chosen to not seek and use healthcare rather than become impoverished [65].

India’s health-financing system is more complex than in other developing countries [13]. The majority of the country’s healthcare is provided by a largely unregulated expensive private sector, which favours the rich [8, 66, 67]. India has one of the world’s highest proportions of OOP payments estimated at 71.1% in 2008–09 [63]. Increased public sector health financing and involvement is critical for improving access to healthcare [68].

Our study shows that lifestyle modification in older adults was predictive of DM medication use in China and India which is consistent with evidence of health promotion programs being implemented in China and India [61, 69]. Rapid economic development in China and India is fuelling increased urbanisation and major societal change. The traditional way of life has been supplanted by modern urban living which is often associated with lower levels of physical activity and unhealthy diets. In addition, aspects of globalisation and industrialization contribute to overweight and obesity and increase the risk of chronic diseases such as DM [45, 58, 61, 70–73]. There is widespread agreement by WHO and other international health authorities that in addition to the use of essential medicines, people with DM should also maintain a healthy lifestyle [74]. Both are concurrent therapies recommended for people with type 2 DM. For example, IDF global guideline for managing older people with type 2 DM recommends that health professionals provide advice and support on lifestyle measures (such as increasing physical activity, stopping smoking and eating a healthy diet) in addition to prescribing appropriate medicines for DM [69].

After adjusting for lifestyle modification and other factors, female sex was a significant predictor of DM medication use in older adults in China. The finding is in line with other epidemiological research on the treatment and control of DM in China [72, 75]. It is suggested, that older women are more likely to seek diagnosis and treatment for DM, for example if they experienced gestational DM during pregnancy, and that men do not pay attention to their health needs to the same extent [38].

The multivariable regression also showed that high BMI and high physical activity were predictors of DM medication use in older men and women in China. An international study of the association between obesity and DM demonstrated that a 10 cm increase in waist circumference and waist-to-height ratio of >0.5 were associated with significant 1.26 (India) and 1.68 (China) times higher odds for DM [76]. Research in China shows that individual’s awareness of having DM increases the likelihood of their undertaking frequent physical activity for self-managing their condition [72]. In India DM commonly occurs at lower obesity thresholds and at younger age compared with many other countries [77, 78]. One theory is that Indian people are genetically predisposed to the development of coronary artery disease which is a risk factor for DM [77].

When holding all other variables constant, association between DM medication use and wealth was positive in India and negative in China. China’s healthcare reforms have increased access to DM medicines among the poor but in India, where there is a dominant private healthcare sector, the rich have better access to DM medicines [8, 66–68]. These findings are consistent with global evidence that in the economically less prosperous developing countries, DM is more prevalent among the rich, but as the pace of economic development increases the association is reversed. Since the 1990s China’s economy has grown faster than that of any other country and this has led to major improvements in income and living standards [7, 12].

China is ahead of India in terms of the implementation of national plans which cover the universal monitoring and surveillance of DM resulting in increased numbers of people being diagnosed and treated [51, 52, 61]. The 2015 the IDF global ranking of absolute numbers of adults (20–79 years) with DM, placed China first (109.6 million) and India second (69.2 million) [16]. However expenditure patterns differ. In 2015 China spent 90 billion International Dollars (ID) on DM-related expenditure, ranking second only to the United States (ID 320 billion). In the same year India spent ID23 billion [16] on DM-related expenditure.

Diabetes medication use was not a significant predictor of catastrophic healthcare expenditure in the presence of lifestyle modification, residence, household wealth, household financial status and the household heads’ educational attainment, in either China or India. In the multivariable analysis in China, rural residents were significantly more likely to experience catastrophic healthcare expenditure compared with urban residents. Yet these results must be interpreted within a broader health policy context. China has made notable progress in expanding government-funded health insurance thereby improving access to medicines and other healthcare across the population. In urban areas health insurance has been extended to the non-employed (e.g., students, the elderly, unemployed) and rural coverage increased from 20% in 2003 to over 85% in 2007 [50]. However there is still a way to go and many people are experiencing catastrophic expenditure due to low levels of paid benefits or subsidies. Our findings show that the proportion of people experiencing catastrophic healthcare expenditure was higher among those in rural areas, with lower education and less wealth.

In the Indian sample large sections of the population still do not have affordable access to healthcare. The country difference can be attributed to interaction between poverty and out-of-pocket (OOP) payments within the context of two very different healthcare systems [10, 50, 79]. Although the probability of catastrophic expenditure is high where poverty levels are high, poverty can result in the exclusion of some sections of the population from healthcare. In this way poverty can provide a somewhat perverse “protection” from catastrophic expenditure [28, 30].

In China older adults with reported DM who were less wealthy were significantly more likely to live in households with catastrophic healthcare expenditure on medications after adjusting for the effects of DM medication use, lifestyle modification, residence, household financial status and household head’s educational attainment. This evidence is consistent with research by Li et al. [30] which showed that a number of factors in combination increase the risk of catastrophic healthcare expenditure. They included being older, having chronic illness and living in rural or socioeconomically deprived areas. Additionally Li et al. [30] suggested that although healthcare utilisation in China has increased due to the expanded breadth in healthcare coverage, low benefit levels are contributing to a higher burdens from OOP payments.

In India older adults with reported DM, with very good or good household financial status, were significantly more likely to live in households with catastrophic healthcare expenditure, compared with their counterparts with moderate, very bad or bad household financial status. This association remained after adjusting for the effects of DM medication use, lifestyle modification, residence, household wealth, and the household head’s educational attainment. However, this does not mean that lower household financial status is protective of catastrophic healthcare expenditure. In this analysis the determinants of catastrophic healthcare expenditure in India need to be understood in the context of equity of access to medication use. In India DM medication use in older adults in the richest wealth quintile was 48.3% compared with 3.9 and 7.2% in the poorest two quintiles. There was also a significant association (*p* < 0.05) between DM medication use and household financial status with 85% of older adults in households with very good or good financial status reporting medication use, compared with 74 and 56% of older adults in households with moderate or very bad household financial status. (Results not shown).

Our findings reflect recent policies and action undertaken in response to the growing burden of DM within the global UHC agenda [61]. Both India and China have committed public funds into healthcare programs for NCDs [29]. China has launched a number of health reforms aimed at improving social health insurance schemes and strengthening primary healthcare [52, 60, 80]. India’s publically-funded health schemes have focused exclusively on inpatient secondary and tertiary care to the neglect of primary healthcare [64] and progress has been impeded by the country’s vast geographic and ethnic diversity [61]. The Indian healthcare system requires a major shift from the traditional paradigm of catering for infectious diseases and maternal and child health, towards primary and secondary prevention, diagnosis, treatment and ensuring the availability and affordability of medications for DM and other NCDs [77, 81].

### Strengths and limitations

We acknowledge the possibility of selection bias because study samples were conditioned on self-reported DM. The use of self-reported measures of chronic disease may substantially underestimate disease prevalence in LMICs, especially within population sub-groups with lower socioeconomic status. An analysis of data from the six SAGE countries found that socioeconomic inequalities in NCD prevalence are more likely to be positive when using self-report compared with symptom-based or criterion-based diagnostic criteria, with greater bias occurring in low-income countries [82].

It is difficult to make accurate comparisons between DM prevalence across studies. Estimates are derived and modelled using a range of methods, definitions, clinical criteria and sampling techniques across geography, demography and time. The purpose of the analysis (e.g., policy or research) is also relevant to the way in which estimates are derived. Nevertheless the quality of WHO-SAGE data is high and these findings are broadly consistent with other major population based studies of DM in China and India.

While every attempt was made to standardise the SAGE survey instruments it is possible that different social and cultural perceptions about DM many have introduced bias. However it is not possible for us to say to what extent this might have occurred.

Other studies have found that people with DM in China and India delay seeking care for financial reasons until after they have developed more serious medical complications [29, 83]. However it is also not possible to ascertain the extent to which this may have occurred here.

The data for this study were cross sectional and therefore causation cannot be assumed in any direction. The analyses cover SAGE Wave 1 data which were collected between 2007 and 2010 in China and India. Future waves of SAGE will enable more wide-ranging analyses of these issues concordant with UHC policies and NCD prevention programmes.

The WHO Study on AGEing and Adult Health (SAGE) provides valid, reliable, comparable national data on important public health outcomes in adults aged 50 and above in China, Ghana, India, Mexico, Russian Federation and South Africa [39]. WHO-SAGE surveys were conducted in the six countries in a highly standardized manner. The questionnaires are first translated into the local language, back translated and validated. WHO-SAGE implemented the quality assurance procedures for household surveys recommended by the United Nations.

WHO-SAGE data have been widely analysed and reported in hundreds of peer-reviewed publications. See http://www.who.int/healthinfo/sage/articles_all/en/. number of such have added to policy evidence by focusing specifically on comparisons between China and India [5, 9, 10, 79]. This study adds to that important body of work.

In the past it has been difficult to make valid cross-country comparisons of catastrophic healthcare expenditures because studies have used a range of variable definitions, expenditure thresholds, study designs, and sampling methods [37]. This is the first study of its kind to use a standardised approach allowing a comparative analysis of data from China and India.

It is more common in studies of this type to include the condition of interest, for example DM, as the independent variable which can mean that undiagnosed cases are erroneously defined as non-cases [29]. Although we have limited our sample size by only including people with reported DM, this allowed an explicit analysis for targeted policy interventions.

## Conclusions

The country comparison reflects major public policy differences underpinned by divergent political and ideological frameworks. China’s expansion of healthcare coverage has increased service access and utilisation but low benefits paid to households have impacted on OOPs. In India healthcare coverage is limited and the government faces ongoing challenges in responding to the health needs of disadvantaged groups in the population. Findings from this study also help reiterate the importance of behavioural factors as essential components of DM management. Health policies and guidelines relevant to DM must therefore incorporate lifestyle modification strategies for effective prevention and control of DM and associated complications. Ensuring equitable and affordable access to medications for DM among older adult populations is fundamental for healthier ageing cohorts and is consistent with the global agenda for UHC.

## Abbreviations

BMI: Body mass index
CI: Confidence interval
DM: Diabetes mellitus
IDF: International Diabetes Federation
KG_s_: Kilograms
LMICs: Low- and middle-income countries
M_s_: Metres
NCDs: Non-communicable diseases
OOP: Out-of-pocket
OR: Odds ratio
PSUs: Primary sampling units
SAGE: Study on global AGEing and adult health
UHC: Universal healthcare coverage
VIF: Variance inflation factor
WHO: World Health Organization
